# Palladium Complex‐Supported Silica Nanocarriers: Enhancing Therapeutic Efficacy and Reducing Curcumin Toxicity in Breast Cancer

**DOI:** 10.1111/jcmm.70757

**Published:** 2025-08-05

**Authors:** Lorenzo Rivas‐García, José Manuel Méndez‐Arriaga, Sandra Jiménez‐Falcao, Paula Sánchez‐Olivares, Pablo Cristóbal‐Cueto, Alejandro Pinedo‐Serrano, Raquel López‐Rosa, Esther Sánchez‐López, Santiago Gómez‐Ruiz, Eva M. Galán‐Moya

**Affiliations:** ^1^ Instituto de Biomedicina (IB‐UCLM) Campus de Albacete, Universidad de Castilla‐La Mancha Albacete Spain; ^2^ COMET‐NANO Group, Departamento de Biología y Geología, Física y Química Inorgánica, ESCET Universidad Rey Juan Carlos Móstoles Madrid Spain; ^3^ Organic Nanotechnology Lab, Departamento de Materiales y Producción Aeroespacial E.T.S.I Aeronáutica y del Espacio Universidad Politécnica de Madrid Madrid Spain; ^4^ Grupo Mixto de Oncología Traslacional UCLM‐GAI Albacete Campus de Albacete, Universidad de Castilla‐La Mancha‐Servicio de Salud de Castilla‐La Mancha Albacete Spain; ^5^ Cirugía General y del Aparato Digestivo, GAI Albacete Servicio de Salud de Castilla‐La Mancha Albacete Spain; ^6^ Facultad de Enfermería Campus de Albacete, Universidad de Castilla‐La Mancha (UCLM) Albacete Spain

**Keywords:** breast cancer, nanomedicine, oncology, organometallic complex, polyphenols

## Abstract

Breast cancer (BC) is the most diagnosed tumour worldwide and triple‐negative breast carcinoma (TNBC) stands out among all subtypes due to its high aggressiveness and high mortality rate. Treatments for this subtype are limited to radio‐and chemotherapy due to the lack of specific therapeutic targets. Nanomedicine emerges as an alternative to overcome actual therapy challenges. Here, we propose a novel nanosystem based on mesoporous silica nanoparticles (MSN) with a curcumin–palladium complex (PdCur) encapsulation that shows promising results against TNBC cells. The resulting nanomaterial, MSN‐AP‐PdCur, displayed an average size of 144 ± 7 nm, determined by transmission electron microscopy and quite good stability through various characterisation techniques. Antitumor activity assays showed a dose‐dependent reduction of the cell metabolic activity of TNBC cells in vitro. Furthermore, the nanosystem mainly favoured the induction of two cell death pathways, such as apoptosis and autophagy, indicating its potential use as a nanomedicine‐based therapeutic agent.

**Abbreviations**
AP: 3‐aminopropyl
ATTCC: American Type Culture Collection
BC: Breast Cancer
BET: Brunauer–Emmett–Teller
BJH: Barret–Joyner–Halenda
CCDP: Cisplatin
CQ: Chloroquine
CTAB: Hexadecyltrimethylammonium bromide
CUR: Curcumin
DCFH‐DA: 2′ ‐ 7′ dichlorofluorescein diacetate
DMEM: Dulbecco's Modified Eagle Medium
DOXO: Doxorubicin
DTX: Docetaxel
FBS: Foetal Bovine Serum
FT‐IR: Fourier transform infrared spectroscopy
H_2_O_2_
: Hydrogen peroxide
IR: Infrared
MSN: Mesoporous Silica Nanoparticles
MSN‐AP: Mesoporous Silica Nanoparticles with (3‐aminopropyl)triethoxysilane
MSN‐AP‐PdCur: Mesoporous Silica Nanoparticles that contain curcumin–palladium complex
MTT: 3‐(4,5‐Dimethylthiazol‐2‐yl)‐2,5‐Diphenyltetrazolium Bromide
PBS: Phosphate Buffered Saline
PC: Phenolic Compounds
PdCur: Curcumin–palladium complex
QVD: Quinolyl‐valyl‐O‐methylaspartyl‐[2,6‐difluorophenoxy]‐methyl ketone
ROS: Reactive Oxygen Species
RT: Room Temperature
SDS‐PAGE: Sodium Dodecyl Sulphate Polyacrylamide Gel Electrophoresis
TBS: Tris‐buffered saline
TEM: Transmission Electronic Microscopy
TEOS: Tetraethyl orthosilicate
TG: Thermogravimetry
TNBC: Triple Negative Breast Cancer
UV: Ultraviolet
XRD: X‐ray diffraction

## 1 Introduction

**FIGURE 1:**
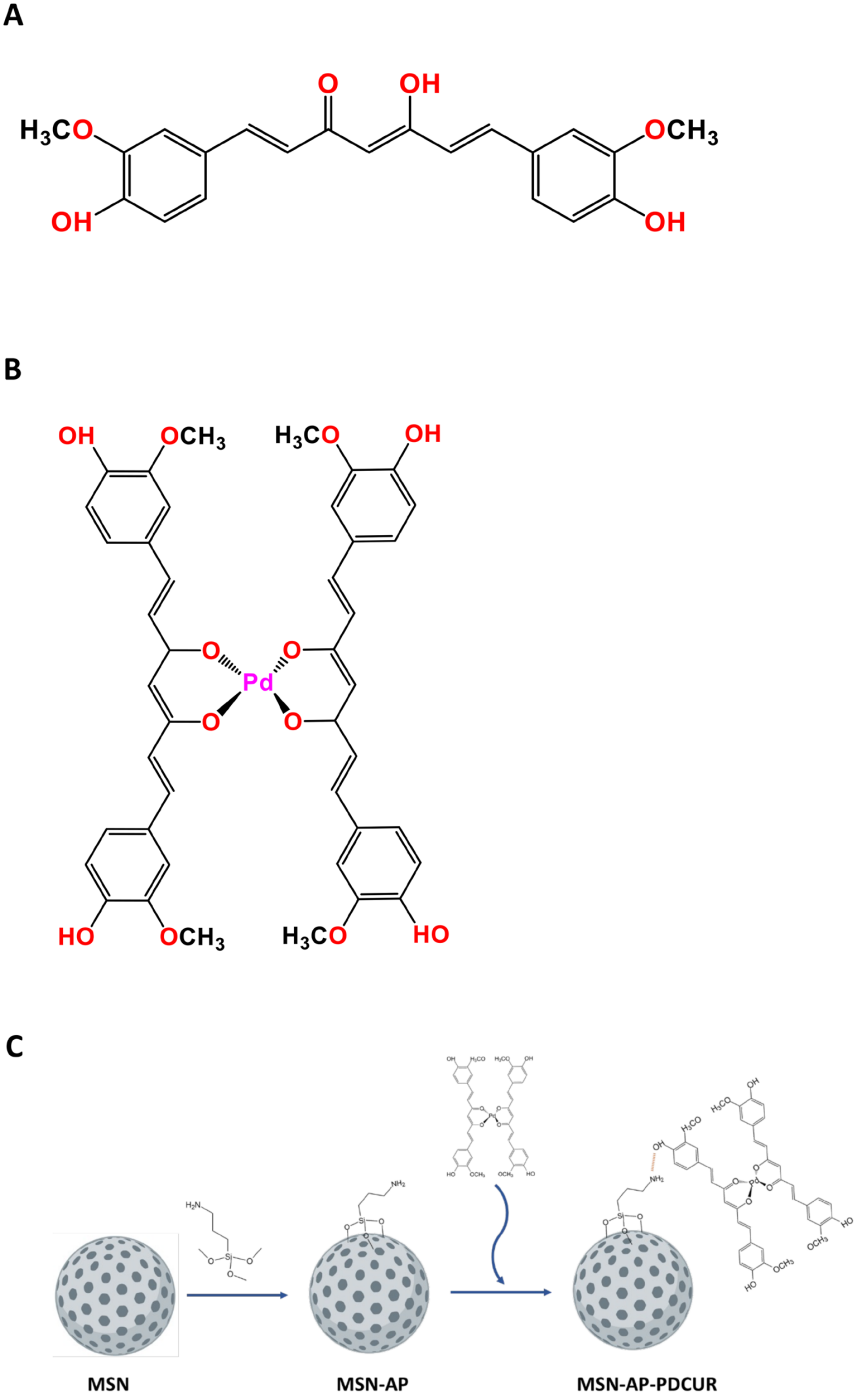
(A) Curcumin structure; (B) PdCur structure (Rodrigues et al. [8]); (C) Reactions of synthesis of PdCur functionalised nanomaterial.

Triple‐negative breast cancer (TNBC) is a type of breast cancer (BC) that lacks the expression of oestrogen receptor, progesterone receptor and human epidermal growth factor receptor 2 (HER2), which makes it unresponsive to targeted therapies commonly used in BC, such as hormonal therapy or the monoclonal antibody trastuzumab [1]. TNBC accounts for approximately 15% of all BC cases and has an earlier onset and higher mortality rate compared to others [1, 2]. Currently, TNBC has limited treatment options, mainly restricted to the use of nonspecific chemotherapeutic agents, such as cisplatin (CCDP), doxorubicin (DOXO) and docetaxel (DTX) [3], generating a need for the development of new therapeutic strategies. Unlike other breast cancer subtypes, TNBC lacks expression of targetable receptors, making it unresponsive to hormone therapy or HER2‐targeted agents. As a result, chemotherapy remains the mainstay of treatment [1]. However, despite initial sensitivity to these cytotoxic agents, TNBC often exhibits rapid disease progression, high recurrence rates and poor overall prognosis [1]. These limitations highlight the urgent need for novel, more effective and less toxic therapeutic strategies [4]. In this context, there has been an increasing interest in organometallic compounds [3] and isolated chemical compounds obtained from plants as therapeutic agents [5, 6]. *Curcuma longa* , commonly named turmeric, is a member of the ginger family that has been used for centuries for medicinal purposes [7]. In recent years, polyphenols obtained from this plant, such as curcumin (Figure 1A), have gained popularity as natural therapeutic agents due to their anti‐inflammatory and antioxidant properties [9, 10, 11] derived from the phenolic compounds (PC). However, curcumin has severe limitations that decrease its bioavailability and therefore its potential use as a therapy. Among its limitations are the limited solubility in water, which prevents it from being administered in aqueous solutions, its fast metabolisation in biological tissues or degradation by other agents such as light, heat or oxygen [12, 13, 14]. For this reason, the encapsulation of curcumin into drug delivery systems might overcome the abovementioned limitations, increasing its bioavailability.

On the other hand, organometallic compounds have shown effective antitumour activity, highlighting platinum‐based compounds such as cisplatin, the main organometallic compound currently used in clinic for the effective treatment of a wide range of cancers [15]. Nevertheless, platinum‐based compounds induce severe toxicological side effects, which could be decreased by the formulation of novel organometallic compounds based on similar metals like palladium. Palladium complexes have emerged as a promising alternative, based on their coordination chemistry similarities with platinum [16, 17, 18] and their demonstrated efficiency against certain cancer types [19, 20].

Concerning all the above, the design of drug delivery systems could reduce side effects while allowing control in the delivery of curcumin and its possible metallic derivatives to target tissues [19], resulting in improved therapeutic efficacy. Mesoporous Silica Nanoparticles (MSN) have been extensively studied in recent years for their potential application in nanomedicine fields such as drug delivery and cancer treatment. Particularly, MSN are effective in delivering natural compounds and isolated chemical compounds obtained from plants [21]. These nanosystems could be synthesised in a variety of shapes and sizes, while their surface based on silanol groups allows the functionalisation with a wide range of drugs, molecules and proteins for specific applications [8]. One of the main advantages of MSN is their high surface area, derived from their large pore sizes ranging from 2 to 50 nm, allowing the encapsulation of large amounts of drug payloads [8]. Encapsulation of active compounds such as curcumin within their pores could improve its low water solubility [21] while keeping the compounds away from the external environment, preventing their degradation or interaction with other tissues until the tumour microenvironment is reached.

This article aims to investigate the potential therapeutic uses of a novel nanosystem based on the cost‐effective synthesis of MSNs loaded with a curcumin–palladium complex (MSN‐AP‐PdCur) against triple negative breast cancer (TNBC).

## 2 Material and Methods

Curcumin–palladium complex (PdCur) was synthesised using K_2_PdCl_4_ (Acros Organics, Massachusetts, USA), curcumin (AlfaAesar, Massachusetts, USA) and triethylamine (VWR Chemicals, Pennsylvania, USA) as received, without additional purification. The reagents used in the preparation of the starting material MSN, namely, hexadecyltrimethylammonium bromide (CTAB) and tetraethyl orthosilicate (TEOS) were purchased from Acros Organics and Sigma Aldrich (Missouri, USA) respectively. 3‐aminopropyltriethoxysilane (AP) from Acros Organics was used for the preparation of the functionalised MSN‐based materials, to improve the adsorption capacity of the nanomaterial. All reactions of silica functionalisation were carried out using standard Schlenk tube techniques in an inert atmosphere of dry nitrogen.

Fourier transform infrared spectroscopy (FT‐IR) spectra were collected using KBr pellets with a spectrophotometer PerkinElmer Spectrum Two. Ultraviolet (UV) spectra were recorded with Jenway 7315 Spectrophotometer equipment. X‐ray diffraction (XRD) patterns were recorded on a Philips Diffractometer model PW3040/00 X'Pert MPD/MRD at 45 kV and 40 mA, using a wavelength of Cu K_α_ (λ = 1.5418). Adsorption–desorption isotherms of nitrogen were measured using a Micromeritics ASAP 2020. The surface areas and the pore sizes were calculated using Brunauer–Emmett–Teller (BET) and Barret–Joyner–Halenda (BJH) methods respectively. Thermogravimetry (TG) analyses were performed with a Shimadzu model at a heating rate of 20°C/min from 30°C to 800°C under nitrogen. Transmission electron microscopy (TEM) images were obtained with a JEOL JEM 1010 at a 100 kV operating voltage. Palladium wt% determinations by X‐ray fluorescence (XRF) were carried out with an X‐ray fluorescence spectrophotometer Philips MagiX with an X‐ray source of 1 kW and an Rh anode using a helium atmosphere. The ICP‐AES study was performed on a Varian Vista AXE Pro Varian 720‐ES (λ_Si_ = 250.69 nm).

### 2.1 Synthesis

#### 2.1.1 Curcumin–Palladium Complex

Metal complex with two curcumin moieties and one Pd atom as the metallic centre (PdCur, Figure 1B) was synthesised following the procedure published by Rodrigues et al. [8]. 0.5 mmol of K_2_PdCl_4_ was dissolved in the minimum amount of water and added to another curcumin solution in 5 mL methanol, heating for 2 h at 60°C under stirring conditions. Then, pH was adjusted to 9.0 by the addition of triethylamine dropwise, keeping the temperature constant and stirring for24 h. The product obtained was recovered by filtration, washing the brown solid powder with methanol and water and dried under vacuum. Infrared (IR), TG and UV spectra confirmed the structure of the palladium complex.

#### 2.1.2 Synthesis of Mesoporous Silica Nanoparticles

The synthesis of MCM‐41 silica nanoparticles (MSN) was performed by the methodology described by Zhao et al. [22] with slight modifications. First, 3.5 mL of a 2 M aqueous sodium hydroxide solution was added to an aqueous solution of CTAB (1.0 g, 2.74 mmol in 480 mL of Milli‐Q water). Subsequently, the silica precursor TEOS (5 mL, 22.4 mmol) was gradually added with vigorous stirring, and the mixture was left to react for 2 h at 80°C. Once the white precipitate arose, the product was filtered and washed using abundant Milli‐Q water and methanol (2 × 20 mL), and dried for 24 h at 80°C. Finally, the resulting MSNs were thermally processed for 24 h at 550°C with an increasing temperature rate of 1°C/min.

#### 2.1.3 Functionalisation of Mesoporous Silica Nanoparticles With (3‐aminopropyl)triethoxysilane (MSN‐AP)

MSN nanoparticles were covered with amino groups from the AP ligand. This reaction was carried out following the procedures reported by our research group [23]. A quantity of 500 mg of MSN was dehydrated at 90°C under vacuum overnight in a Schlenk tube. The activated material was then dispersed in 40 mL of dry toluene, adding 525 μL of AP (100% w/w AP/SiO_2_) to the solution. The mixture was heated up to 110°C and stirred for 48 h. Finally, the dispersion was centrifuged, and the solid was washed twice with toluene and once with diethyl ether. The resulting white solid, MSN‐AP, was dried overnight at 70°C on a stove.

#### 2.1.4 Palladium–Curcumin Complex Adsorption (MSN‐AP‐PdCur)

PdCur complex was incorporated into the silica nanomaterial MSN by adsorption method. Initially, 100 mg of MSN‐AP was dispersed in 10 mL of ethanol. After 15 min of vigorous stirring, 20 mg of PdCur were added to obtain a 1:5 proportion between the complex and silica in the final material MSN‐AP‐PdCur. The suspension was stirred for 48 h at room temperature (RT). Finally, the mixture was centrifuged, and the isolated solid was washed with ethanol once and dried under a vacuum. The resulting nanomaterials were stored at room temperature. The complex molecules were adsorbed onto the nanomaterial surface by hydrogen bonding between amino groups from the linker and the hydroxyl groups of the palladium complex. Wt% of incorporated PdCur complex was calculated with thermogravimetric analysis (Section 3.1.5).

### 2.2 Antitumoral In Vitro Studies

#### 2.2.1 Cell Culture Conditions

MDA‐MB‐231 and MCF10A cells were purchased from American Type Culture Collection (ATCC). Cells were maintained in Dulbecco's Modified Eagle Medium (DMEM) supplemented with 10% (*v/v*) foetal bovine serum (FBS), 100 U/mL penicillin, 100 μg/mL streptomycin and 2 mM L‐glutamine (all reagents from Sigma Aldrich, Missouri, USA). Cells were kept at 37°C in a humidified atmosphere of 5% CO_2_, and the medium was replaced every 2–3 days. Upon reaching confluence, cells were treated with trypsin–EDTA solution (Sigma Aldrich, St Louis, USA). MCF10A cells were cultured employing the procedure previously described [24].

#### 2.2.2 MTT Assay

Cells were seeded in 48‐multiwell plates at a density of 1 × 10^4^ cells per well. After 24 h, the culture medium was replaced and the cells were treated with MSN‐AP‐PdCur and curcumin alone (CUR) at 5, 10, 15 and 20 μg/mL of curcumin concentration for 72 h. After this time, the medium was retired and substituted by a 0.5 mg/mL solution of MTT ((3‐(4,5‐Dimethylthiazol‐2‐yl)‐2,5‐Diphenyltetrazolium Bromide), Sigma Aldrich, Missouri, USA) prepared in PBS (Phosphate Buffered Saline, Sigma Aldrich, Missouri, USA) for 1 h at 37°C. Then, the MTT was removed and DMSO was added. Finally, absorbance values were measured at 555 nm, using 690 nm as reference wavelength, in a plate reader (BMG Labtech, Ortenberg, Germany). Then, the IC_50_ concentration was determined based on viability‐concentration curves using GraphPad Prism 8 software.

Alternatively, the effects of several reference therapeutic agents were evaluated to compare them with the nanosystem‐mediated effect. Increasing doses of CCDP, DTX and DOXO were administered during a 72‐h exposure period.

#### 2.2.3 Cell Death Mechanism

##### 2.2.3.1 Apoptosis

Cells were treated with IC_50_ of MSN‐AP‐PdCur for 72 h. Then, supernatants and cells were collected and washed twice with cold PBS. Subsequently, cells were stained with Annexin V‐DT‐634 (Immunostep S.L., Salamanca, Spain) and 3 μL of PI (10 mg/mL) in 1 × Binding Buffer (10 mM HEPES, pH 7.4, 140 mM NaOH, 2.5 mM CaCl_2_) at room temperature for 1 h under dark conditions. Stained cells were analysed using a FACSCanto II flow cytometer (BD Biosciences, San Jose, CA, USA). Early (Annexin V‐positive, PI‐negative) and late (Annexin V‐positive and PI‐positive) apoptotic cells were included in the determination of cell death. Furthermore, the % of living cells, early and late apoptotic cells and necrotic cells were included. Data are presented as the mean of three independent experiments.

For apoptosis inhibition, TNBC cells were pretreated with 10 μM QVD (quinolyl‐valyl‐O‐methylaspartyl‐[2,6‐difluorophenoxy]‐methyl ketone) for 45 min. Subsequently, the cells were co‐incubated with 5 μM QVD and IC_50_ MSN‐AP‐PdCur for 72 h.

##### 2.2.3.2 Autophagy

Cells were treated with IC_50_ of MSN‐AP‐PdCur for 72 h. Afterwards, cells were detached, and the autophagy induction was analysed by flow cytometry using the Autophagy Detection Kit 2.0 (Enzo Life Sciences, Switzerland) following the manufacturer's instructions. Rapamycin was the positive control (500 nM for 16 h, Sigma Aldrich, Missouri, USA).

For autophagy induction, TNBC cells were treated with chloroquine (CQ) 50 μM for 12 h.

#### 2.2.4 Western Blot

Western blot preparation was carried out following the procedure proposed previously [25]. We used the following primary human monoclonal antibodies: anti‐LC3B (Santa Cruz Biotechnology, CA, USA), anti‐PH2AX (Santa Cruz Biotechnology, CA, USA), anti‐GAPDH (Santa Cruz Biotechnology, CA, USA) and anti‐pS6 (Cell Signalling Technologies, Beverly, MA, USA). Horseradish peroxidase‐coupled secondary antibodies (anti‐rabbit 1:10.000, or anti‐mouse 1:5.000) were used to detect the protein‐bound primary antibodies. Protein bands were exposed using the ECL Plus Western Blotting Detection System (GE Healthcare, UK).

#### 2.2.5 ROS Production

Cells were treated with IC_50_ of MSN‐AP‐PdCur for 72 h. After incubation, the medium was retired, and cells were incubated with 50 μM 2′‐7′ dichlorofluorescein diacetate (DCFH‐DA) at 37°C for 30 min. Subsequently, DCFH‐DA was removed, and cells were washed with PBS. The induction of ROS was measured by flow cytometry using a FACSCanto II flow cytometer (BD Biosciences, San Jose, CA, USA). Hydrogen peroxide (H_2_O_2_) 25 μM was employed as positive control. Data are presented as the mean of three independent experiments.

### 2.3 Statistical Analysis

For in vitro studies, descriptive statistical parameters (means and standard error of the mean (SEM) of the experiment) were obtained for all the variables studied. The normal distribution of the variables was studied by the Kolmogorov–Smirnov test. Additionally, the homogeneity of variance was examined with the Levene test. The differences among the groups were evaluated by Student's t‐test or ANOVA test for normally distributed variables, and the Mann–Whitney‐U test was used for non‐normally distributed variables. Statistical significance was considered at *p* < 0.05. All the experiments were performed in triplicate. GraphPad Prism 8 software was used to perform the graphics.

## 3 Results and Discussion

The PdCur loaded silica material was synthesised using MCM‐41 mesoporous silica (MSN) as the host material (Figure 1C). Although porous materials can usually be loaded by stirring in a saturated solution of the therapeutic cargo, the presence of amine groups provided by AP moieties facilitates the formation of weak intermolecular forces with the polar groups of curcumin ligands. Hydrogen bonds between the amino group and hydroxyl groups prove to be critical for adsorption and controlled release of the drug into the culture medium, as previously demonstrated in other amino‐functionalised systems reported [26, 27]. Grafting reactions of AP with MSN were used to synthesise the amine functionalised material MSN‐AP (Figure 1C). The last step was the loading of the palladium complex. MSN‐AP was refluxed with the curcumin–palladium complex in ethanol for 48 h under nitrogen atmosphere to form the curcumin complex loaded amine functionalised mesoporous silica material MSN‐AP‐PdCur, in a theoretical 20 wt% ratio (Figure 1C). This nanomaterial was then filtered and dried for 24 h under vacuum at RT.

Large‐scale production of functionalised silica nanoparticles is increasingly feasible due to advancements in synthesis methods and the availability of raw materials [28]. Techniques such as sol–gel processes, flame synthesis and hydrothermal methods have been optimised to produce high‐quality nanoparticles efficiently. However, several challenges remain in clinical translation, like ensuring the long‐term stability of functionalised silica nanoparticles or developing advanced coatings to enhance biocompatibility and reduce immune system detection. One of the advantages of these nanomaterials is the functionalisation possibilities [26, 27], which are potentially unlimited. Once an optimal linker capable of bonding covalently to the silica surface is established, it is possible to include any desired functional group, directed to the exterior area of the nanomaterial to act as a key for molecule or biomolecule targets or as a reactive group for further chemical reactions.

Focusing on palladium complexes, there are few examples in which curcumin is used as a ligand, like the previous work developed by Rodrigues et al. [8] in which the studied Pd complex is based Additionally, there are many other examples of palladium with curcuminoids for anticancer applications [29, 30].

### 3.1 Nanoparticle Characterisation

#### 3.1.1 TEM

**FIGURE 2:**
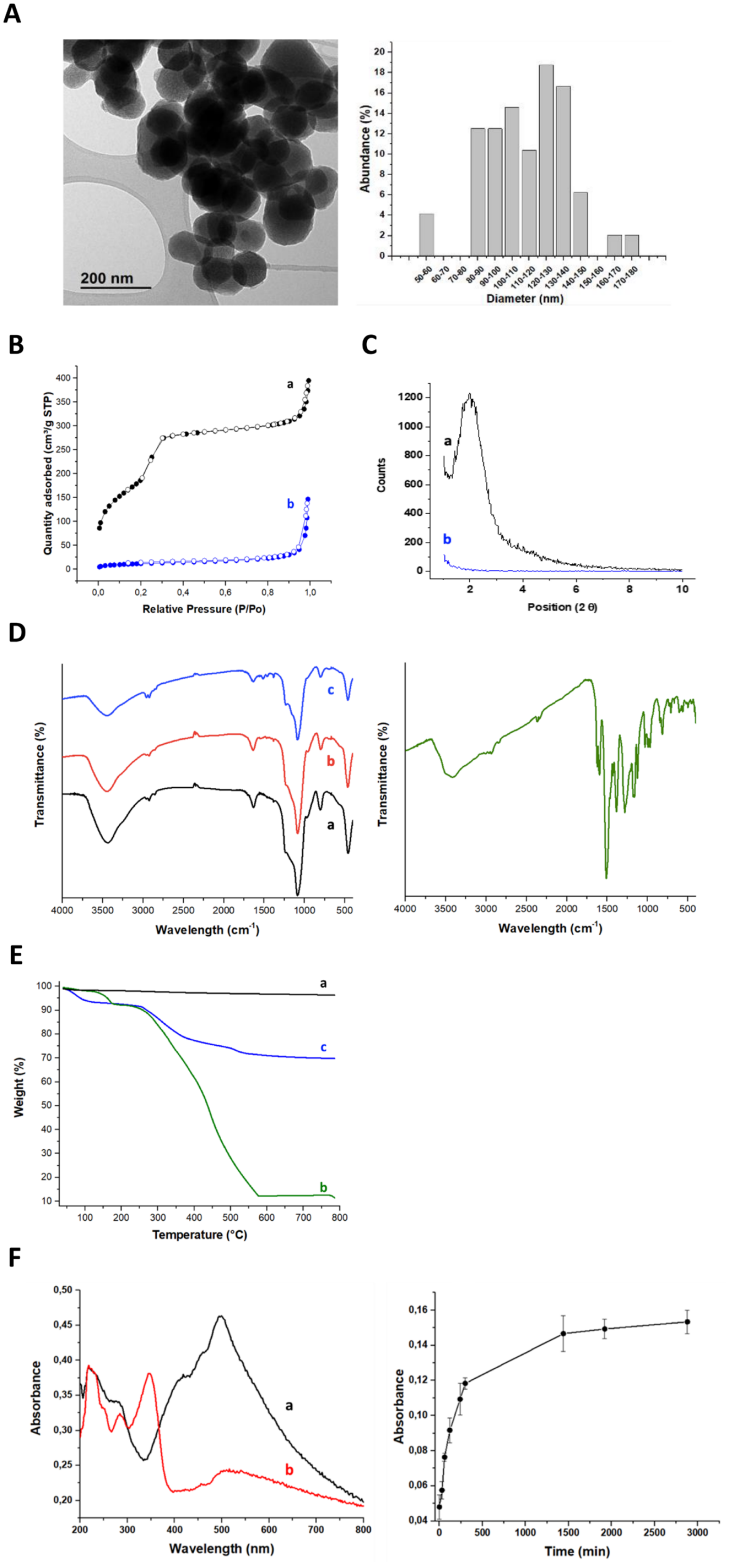
Characterisation of the synthesised nanosystem. (A) MSN TEM micrography and mean diameter size distribution of MSN. (B) N_2_ adsorption/desorption isotherms of MSN (a) and MSN‐AP‐PdCur (b); (C) XRD diffraction patterns of MSN (a) and MSN‐AP‐PdCur (b); (D) FT‐IR spectra from each isolated material for the preparation of MSN‐AP‐PdCur (MSN [a], MSN‐AP [b] and MSN‐AP‐PdCur [c]) and PdCur complex; (E) Thermogravimetric decomposition profile of MSN (a), PdCur (b) and MSN‐AP‐PdCur (c); (F) 1‐ UV–Vis spectra of the PdCur complex in PBS (1 mg mL‐1) freshly dissolved (a) and incubated at 37°C for 24 h (b). 2‐ Kinetic release profile of the PdCur complex from MSN‐AP‐PdCur.

The starting material MSN was characterised by TEM. As shown in Figure 2A, MSN presents a semispherical appearance with a narrow size distribution and an average diameter of 114 ± 7 nm. In addition, well‐defined hexagonal pores can be noted, which are typical of MCM‐41 MSN.

#### 3.1.2 BET

To characterise the porous properties of the starting material and the functionalised carrier, nitrogen adsorption/desorption isotherms were analysed at 77 K for both MSN and MSN‐AP‐PdCur, as shown in Figure 2B. Experimental results make evident a decrease in the surface area of the modified MSN compared to the starting material. MSN exhibits the expected type IV isotherm (characteristic of mesoporous silica supports), while MSN‐AP‐PdCur shows the characteristic appearance of materials with filled pores [31, 32]. Data fitting of the nitrogen adsorption/desorption results to Brunauer–Emmett–Teller (BET) isotherms permitted the determination of the most remarkable surface parameters (Table 1). The raw MSN presented a BET surface of 682 m^2^g^−1^, a pore volume of 0.52 cm^3^g^−1^ and a 3,2 nm pore diameter. The functionalisation of the MSN surface with AP and the subsequent adhesion of the PdCur complex resulted in a decrease in the BET surface, the pore volume and the pore diameter to 42 m^2^g^−1^, a 0.11 cm^3^g^−1^ and 1.0 nm respectively.

**TABLE 1:** Surface parameters of MSN and MSN‐AP‐PdCur obtained by N2 adsorption/desorption analysis.

Material	S_BET_ (m^2^g^−1^)	Total pore volume (cm^3^g^−1^)	Pore diameter (nm)
MSN	682	0.52	3, 2
MSN‐AP‐PdCur	42	0.11	1, 0

#### 3.1.3 DRX

The starting MSN and the final material loaded with PdCur complex (MSN‐AP‐PdCur) were further characterised by powder X‐ray diffraction measurements (Figure 2C). A hexagonal pore distribution expected for mesoporous silica is present in the MSN nanosystem [33, 34]. The diffraction peak associated with the Miller plane (100) is observed at ca. 2.01° in MSN material, and it disappears in the MSN‐AP‐PdCur pattern, confirming the occupancy of the mentioned pores.

#### 3.1.4 FT‐IR

FT‐IR analysis confirmed the adequate functionalisation of the complex (Figure 2D). The MSN starting material presented the typical signals associated with siliceous materials: a peak at 464, 805, 970 cm^−1^ and a sharp and strong band at 1077 cm^−1^, corresponding to the Si‐O, SiO_4_, Si‐OH and Si‐O‐Si vibrations respectively [35]. Besides, the free silanol groups and adsorbed water onto the material cause a typical broadband between 3000 and 3750 cm^−1^ associated with the O‐H bond. The following isolated material, MSN‐AP, presented a very similar FT‐IR pattern, except for the presence of two small broad bands at 1640 and 695 cm^−1^ caused by the N‐H bond [36]. Finally, the PdCur complex inclusion into the MSN (MSN‐Ap‐PdCur) carrier was confirmed by the appearance of several peaks between 1500 and 1200 cm^−1^.

The PdCur complex identity was also confirmed by FT‐IR, owing to the presence of the characteristic signals from curcumin. Signals present from 3085 to 2800 cm^−1^ are originated by O‐H, C‐H (both aromatic and aliphatic) bonds. Other remarkable signals found at lower wavenumbers are those proceeding from the overlying C=O and C=C aliphatic bonds (located at 1629 cm^−1^), C=C aromatic and C=O vibrations (which appear at 1500 cm^−1^) and C‐O phenolic bond (resulting in a strong band at 1269 cm^−1^) [8, 37].

#### 3.1.5 TG

Thermogravimetric (TG) analysis evaluated the thermal stability of each intermediate and calculated the rate of functionalisation after each step. As can be seen in Figure 2E, MSN is highly stable, since its mass loss is negligible until the highest temperature analysed. On the contrary, PdCur has shown to be much more sensitive to high temperatures, especially above 300°C. It exhibits a first thermal transition at 160°C (mass loss 6%) and a second thermal decomposition which occurs in two steps with maximum decomposition rates at 340°C and 482°C respectively. This second thermal decomposition represents an 80% mass loss, attributed to the curcumin ligand. Nevertheless, it seems that a mild thermal stabilisation is happening due to the experimental decomposition temperature of 580°, while according to the bibliography it should be 500°C approximately [38]. The calculated amount of Pd in the PdCur complex is 15%, a percentage also confirmed by ICP and FRX techniques. On the other hand, the TG decomposition profile of MSN‐AP‐PdCur shows a first thermal transition below 100°C related to the water absorbed, while the thermal decomposition of the PdCur complex indicates a composition of 24% in MSN‐AP‐PdCur.

### 3.2 PdCur Release

To elucidate the release profile of PdCur from the MSN, a specific release assay was conducted. Thus, 5 mg of MSN‐AP‐PdCur were suspended in 5 mL of PBS buffer. This buffer was chosen for being a similar option to the cell culture media that was subsequently used. After an initial sonication, the sample was incubated at 37°C and centrifuged at specific time intervals to analyse the absorption. Although the most intuitive wavelength to be chosen according to the UV–VIS spectra of MSN‐AP‐PdCur was 500 nm (Figure 2F), no increase was observed over time at 500 nm. In spite, it was detected a rapid growth in the signal at 340 nm in the first 300 min (5 h), which was gradually stabilised in the following measurements (300–3000 min [2 days]), as shown in Figure 2F. The change in the PdCur absorption profile along time (Figure 2F) could be attributed to the hydrolysis of the PdCur and subsequent substitution of the curcumin by the hydroxyl groups. In that way, MSN are prone to aggregation and degradation, which can affect their release properties and effectiveness as drug carriers, but as it has seen in bibliography, MSN have proven to be effective in delivering natural compounds and isolated chemical molecules as it happens with curcumin [39].

Despite no specific pharmacokinetic studies having been performed in this study, it is worth mentioning that the behaviour expected for the PdCur complex here proposed will be different from free curcumin [40, 41]. On the one hand, two molecules of curcumin are incorporated into the palladium complex, resulting not only in a bigger molecule but in a molecule with different chemical properties than free curcumin. As can be found in the literature, stability, solubility and pharmacodynamic effects are better for curcumin metal complexes than for free curcumin. On the other hand, this Pd metal complex is anchored onto a mesoporous silica nanoparticle that will be passively delivered into the tumour by the enhanced permeability and retention effect due to the potential accumulation of nanoparticles, which would enhance the potential uptake of the therapeutic species by cancer cells.

### 3.3 In Vitro Antitumoral Activity

Firstly, to test the antitumoral capacity of the nanosystem, an MTT test was conducted. An incubation time of 72 h was selected for being the time when the release capacity of the nanosystem reached the maximum peak. According to Figure 2F, the release of the PdCur complex was maximum at 3000 min; thus, at 4320 min (72 h), after reaching the maximum release, the potential effect on cell viability should be observed.

Firstly, each component of the nanosystem was evaluated individually. Both isolated curcumin and the MSN‐AP‐PdCur nanosystem significantly reduced cell metabolism, whereas the other components showed no noticeable effect on MTT metabolisation (data not shown). This suggests that antitumour activity was primarily mediated by curcumin, with the nanosystem MSN‐AP‐PdCur working as an effective delivery vehicle.

**FIGURE 3:**
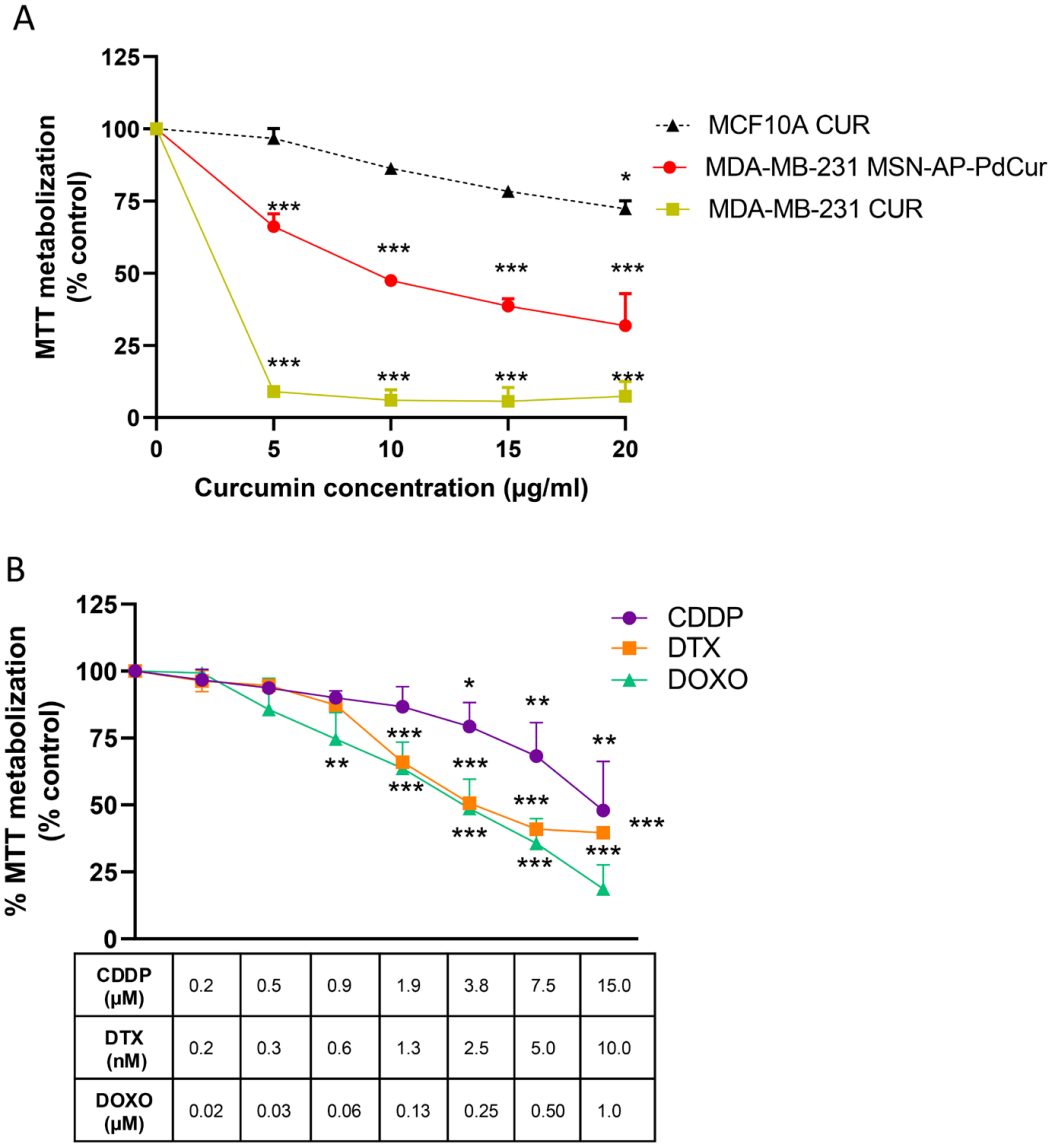
MTT metabolisation assays after 72 h of different treatments. (A) MTT metabolisation in breast cancer cell line MDA‐MB‐231 and MCF10A cells after 72 h of treatment with MSN‐AP‐PdCur and curcumin (CUR); (B) MTT metabolisation in MDA‐MB‐231 after 72 h incubation with different doses of DOXO, CDDP and DTX. Groups were compared with control cells (untreated cells). T‐test *p < 0.05; **p < 0.01; ***p < 0.001. *n* = 3.

Notably, under these conditions, a dose‐dependent response was observed only for the MSN‐AP‐PdCur nanosystem, but not for the isolated curcumin (Figure 3A). In fact, treatment of MDA‐MB‐231 TNBC cells with this PC isolated for 72 h led to a consistent reduction in MTT cell metabolism by approximately 90% across all the tested doses, with a calculated IC_50_ of 1.89 μg/mL. These results are partially consistent with previous studies, which reported an IC_50_ of approximately 30 μg/mL for isolated curcumin after 48 h of incubation [42, 43]. In our experimental setup, at lower doses than reported in the literature but at longer times, we observed an increase in the reduction of cell viability by curcumin, which confirms its potent antitumour effect. In order to overcome pharmacokinetic challenges such as poor absorption and rapid metabolisation of curcumin, we generated an organometallic compound based on palladium and curcumin, and we encapsulated it into the MSN structure. The result of exposing a TNBC tumour line to this nanosystem showed a dose‐dependent decrease in MTT metabolism, offering a controlled and effective delivery system. Figure 3A shows that when loaded in the MSN‐AP‐PdCur, the lowest dose of curcumin (5 μg/mL) resulted in a reduction rate of about 40%, and the highest (20 μg/mL) decreased cell metabolic activity up to 70%. The IC_50_ value of this nanosystem was 9.4 μg/mL. Notably, no significant cytotoxic effects were observed on the nontumorigenic epithelial cell line MCF10A at a concentration of 5 μg/mL. In fact, only the highest concentration of the nanosystem (20 μg/mL) produced a statistically significant reduction in cell viability, which was around 25%. This effect was nearly three times lower than observed in TNBC cells at the same concentration, highlighting the compound's selective cytotoxicity towards malignant breast cells.

Previous studies have employed nanotechnological strategies to encapsulate curcumin, highlighting that the loading capacity of the nanostructure is a critical factor in determining its potential as an antitumour agent. For instance, the drug loading capacity significantly influenced the IC_50_ values of a polymeric nanoparticle composed of poly‐glycerol‐malic acid‐dodecanedioic acid encapsulating curcumin. The nanostructures that contained more curcumin exhibited a lower IC_50_. Particularly, the IC_50_ values were 40.2 and 33.6 μM at 48 h for curcumin nanoparticles 7:3 and curcumin nanoparticles 6:4 respectively [44]. Generally, nanostructures that have a higher drug capacity, such as polymeric or organic nanoparticles [45, 46, 47], tended to have lower IC_50_ values compared to other types of nanosystems with poor loading capacity, such as inorganic nanoparticles that utilised gold [48] or iron [49] nanoparticles as drug carriers. This suggests that optimising the curcumin payload within nanocarriers is essential for enhancing its therapeutic efficacy. Under the present conditions, two changes were observed. Our MSN‐AP‐PdCur reported the highest curcumin loading capacity compared to other nanomaterials (e.g., Chen P.C. described a curcumin encapsulation into a polymeric nanoparticle using an oil‐in‐water emulsion with a drug capacity of 32% [45], and MSN‐AP‐PdCur described a drug capacity charge about 50%), and our MSN‐AP‐PdCur had the lowest IC_50_ value against the MDA‐MB‐231 cancer cell line, compared to other solutions proposed to improve the curcumin delivery based on nanotechnology [45, 48]. These records make MSN‐AP‐PdCur one of the most effective drug delivery systems for curcumin.

Next, the effects of three chemotherapeutic agents used as gold standards in TNBC treatment, namely, CDDP, DTX and DOXO were evaluated for comparative purposes (Figure 3B). The IC₅₀ values obtained for these reference compounds were lower than those of MSN‐AP‐PdCur (Table 2), indicating higher in vitro cytotoxic potency in MDA‐MB‐231 cells. Thus, CDDP reported IC_50_ 4.2 μg/mL, DTX 0.00485 μg/mL, and DOX 0.23 μg/mL (Table 2).

**TABLE 2:** IC_50_ against MDA‐MB‐231 of MSN‐AP‐PdCur, CCDP, DOXO and DTX.

Therapeutic agent	IC_50_ (μg/mL)
MSN‐AP‐PdCur	9.4
CCDP	4.20
DOXO	0.234
DTX	0.00485

However, it is important to note that conventional chemotherapeutics are often associated with significant systemic toxicity. In contrast, the MSN‐AP‐PdCur nanosystem, despite exhibiting a higher IC_50_, offers several potential advantages such as enhanced biocompatibility and reduced off‐target effects. Toxicity associated with all of these first‐line chemotherapeutic agents on nontumour cells has been previously reported [50, 51, 52]. For example, repeated treatment with CCDP has been shown to induce senescence in fibroblasts [50]. This represents one of the main advantages of this nanocomposite, which, at therapeutic doses, does not affect MTT metabolism in nontumorigenic cells (MCF10A).

Furthermore, the incorporation of curcumin and palladium within a mesoporous silica carrier may provide added benefits related to controlled release and stability. Altogether, these results allow us to position our nanosystem within the broader landscape of cancer treatment strategies.

One of the main limitations in the potential application of curcumin is its poor bioavailability, which stems from issues related to dosing and release. The use of nanoparticles offers a promising strategy to overcome these challenges by enabling better handling and controlled delivery. Several studies have demonstrated that nanoparticle inclusion enhances the bioavailability of curcumin in various cell types, including lung cells, and may also help minimise its toxic effects [53, 54].

However, direct comparisons are challenging due to the novelty of combining palladium (Pd) and curcumin in a single nanosystem. As summarised in a recent review by Ghoran et al. [55], a wide range of nanosystems have been developed for curcumin delivery, including liposomes, polymeric nanoparticles and mesoporous silica‐based platforms. Furthermore, Pd–curcumin complexes have shown promising anticancer activity, as reported by Dutra et al. [29].

To the best of our knowledge, no previous reports have described the integration of Pd–curcumin complexes within a mesoporous silica nanoparticle framework, as achieved in our MSN‐AP‐PdCur formulation. This suggests that our system represents a novel nanoformulation strategy that combines the therapeutic advantages of both Pd–curcumin complexes and silica‐based nanocarriers.

**FIGURE 4:**
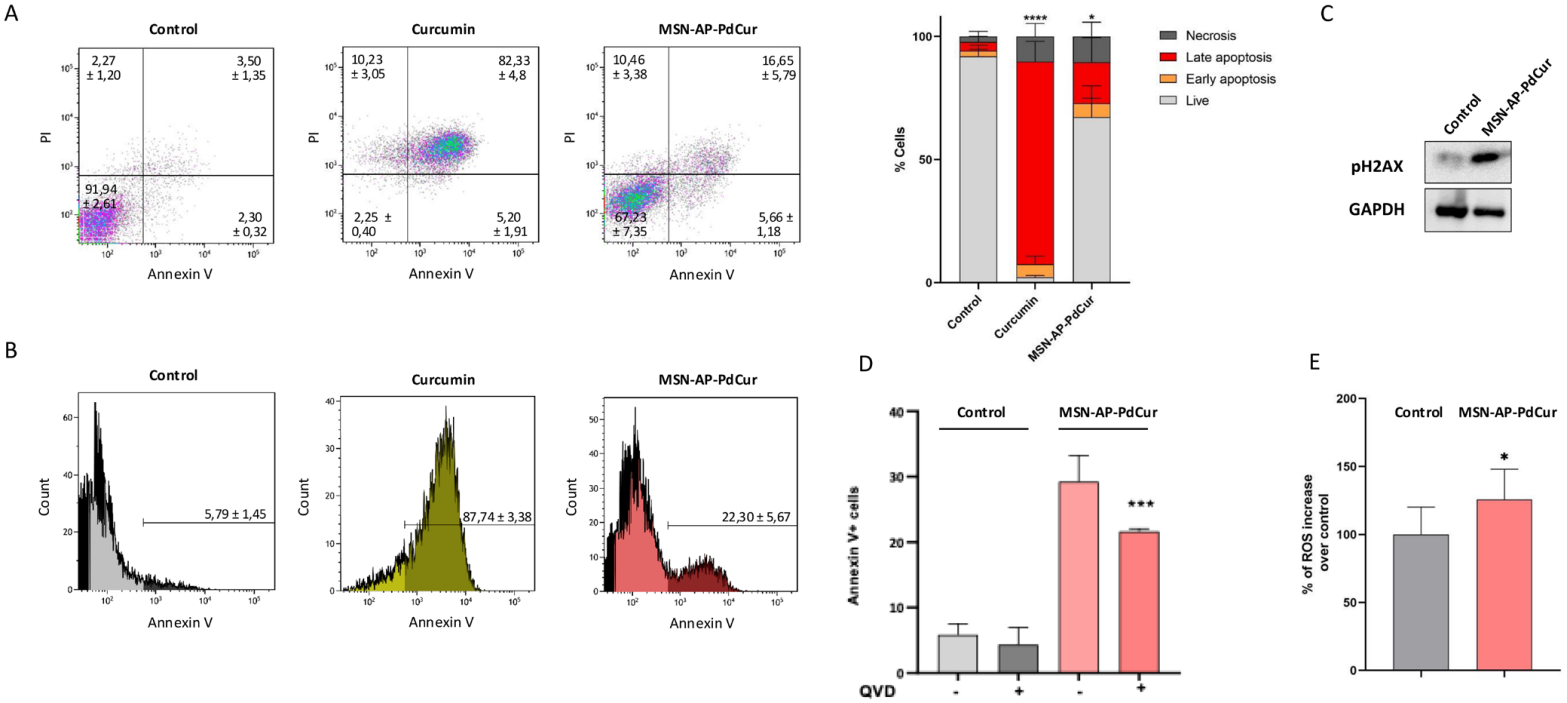
Effect of the incubation of MDA‐MB‐231 cells with IC_50_ of MSN‐AP‐PdCur for 72 h in the apoptotic process measured by flow cytometry. (A) Dot‐plot representation and percentage of live cells (AV‐/PI‐), cells in early apoptosis (AV+/PI‐), cells in late apoptosis (AV+/PI+) and necrotic cells (AV‐/PI+); (B) Histogram of Annexin V positive cells (C) Protein expression of PH2AX after 72‐h treatment with the IC_50_ of MSN‐AP‐PdCur evaluated by Western blot. GAPDH was the loading control. (D) Annexin V positive cells with cells incubated and nonincubated with QVD; (E) ROS production. H_2_O_2_ 25 μM was employed as a positive control. Mean ± SEM. *p < 0.05; *** < 0.001. t‐test; *n* = 3.

Subsequently, the mechanisms of cell death associated with MSN‐AP‐PdCur were studied using several tests. Figure 4A and Figure 4B show the impact of the incubation with MSN‐AP‐PdCur on the induction of apoptosis in TNBC cells using flow cytometry. Exposition to this nanosystem induced an increase in the rates of early and late apoptosis at 72 h and, therefore, significant changes were observed in the amount of living cells in response to MSN‐AP‐PdCur (Figure 4A, right panel). Moreover, the exposition to the nanosystem significantly augmented the % of apoptotic cells as measured by annexin V positive cells (Figure 4B, right panel). Additionally, to confirm the role of MSN‐AP‐PdCur in the apoptotic process, the protein expression of Phospho‐Histone H2AX was measured. In the apoptosis process, H2AX phosphorylation is an early event that initiates the cascade of events leading to cell death [56]. Under the present conditions, the treatment of TNBC cells with MSN‐AP‐PdCur increased the phosphorylation of H2AX (Figure 4C). H2AX plays a role in DNA repair and the maintenance of genomic stability. Its phosphorylation is a marker of DNA damage and is necessary for the recruitment of repair proteins to the site of damage. Currently, the excessive toxicity and challenges in dosage management associated with curcumin limit its potential therapeutic applications. For example, treatment of TNBC cells with the IC_50_ of isolated curcumin resulted in such high toxicity that it was not possible to collect sufficient samples to assess changes in H2AX phosphorylation, which underscores curcumin's extreme cytotoxicity. In contrast, treatment with the IC_50_ of MSN‐AP‐PdCur enables a controlled and sequential release of curcumin, as evidenced by increased H2AX phosphorylation after 72 h, with expected further apoptotic activity with prolonged exposure. Therefore, incorporating curcumin into this nanosystem provides controlled release and enhances its therapeutic potential.

Next, to assess whether the apoptosis induced by the nanoparticle was caspase‐dependent, the pan‐caspase inhibitor QVD‐Oph (QVD) [57] was employed. Treatment with QVD partially inhibited the apoptotic effect of MSN‐AP‐PdCur in TNBC cells (Figure 4D), as evidenced by a significant reduction in Annexin V‐positive cells. These findings suggest that caspases are involved in the pro‐apoptotic mechanism triggered by the nanosystem.

This set of results shows how MSN‐AP‐PdCur activates apoptotic processes in TNBC cells, which is in agreement with other authors who have shown this apoptotic activity in curcumin (alone or encapsulated into various NPs types) in cancer cells [42, 44, 58]. Bare curcumin has been shown to induce an apoptotic antiproliferative effect mediated by the activation of caspase‐3 and caspase‐9 [59] while its encapsulation into a nanosystem as a carrier replicates this ability to induce apoptosis. Our results indicate that MSN‐AP‐PdCur also induces caspase‐dependent apoptosis, as evidenced by the partial reversal of Annexin V‐positive cell levels upon treatment with the pan‐caspase inhibitor QVD. Moreover, curcumin embedded in inorganic carriers has been shown to enhance the expression of cleaved caspase‐3 and modulate the BAX/Bcl2 ratio [49], offering an effective antiproliferative strategy. This approach allows the combination of curcumin's therapeutic effects with other drugs, further amplifying its anticancer potential [60, 61].

**FIGURE 5:**
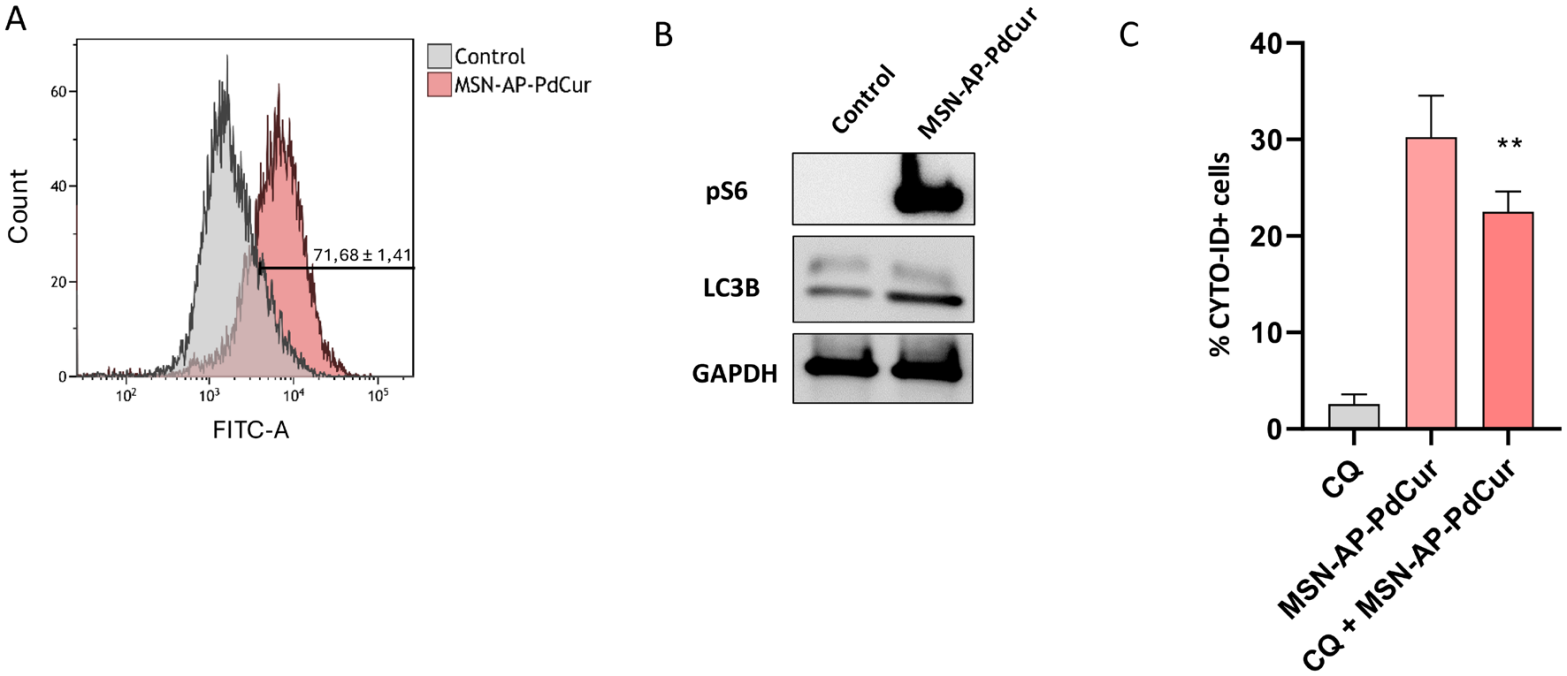
Effect of the incubation of MDA‐MB‐231 cells with IC_50_ of MSN‐AP‐PdCur for 72 h (A) autophagy induction by flow cytometry; (B) Expression of pS6, PH2AC and LC3‐B after 72‐h treatment with the IC_50_ of MSN‐AP‐PdCur by Western blot. GAPDH was the loading control; (C) Percentage of CYTDO‐ID+ cells after the treatment with 50 μM CQ for 12 h. **p < 0.01; ***p < 0.001. t‐test; *n* = 3.

As seen in this work, treatment with curcumin embedded in polymeric nanoparticles induces up to 30% of apoptosis in tumoral cells, a moderated apoptotic response that, although not as high as it happens in other nanocarrier systems, is still significant for the treatment of TNBC cells. Some authors propose that the main role of PC in cancer treatment is mediated by excessive ROS production that could trigger apoptosis and other cell death mechanisms [62]. Thus, the effect of MSN‐AP‐PdCur to induce oxidative stress was investigated, showing a significant increase of ROS production upon exposure to the nanosystem for 72 h (Figure 4E). This fact was consistent with other authors’ observations describing oxidative damage from curcumin and other phenolic compounds [5, 6]. Lastly, autophagy, well known as a cellular process involved in degradation and recycling cellular components through the lysosomal system [51], plays a key role in cellular homeostasis and can be activated in response to a variety of stressful conditions such as nutrient deprivation and oxidative stress [51]. For this reason, we have studied the potential of MSN‐AP‐PdCur to induce autophagy in TNBC cells. MSN‐AP‐PdCur generates high levels of autophagy when in contact with TNBC cells, as is shown in flow cytometry measurements (Figure 5A). In cancer, autophagy's activation can either have a pro‐survival or pro‐death role depending on the context, but at high levels, it can contribute to cell death [52].

Subsequently, this molecular mechanism of cell death associated with MSN‐AP‐PdCur was confirmed by Western blot assays. These assays show phosphorylation of S6, a protein involved in cell growth and proliferation that is phosphorylated upon autophagy activation; and the conversion ratio of LC3B‐I to LC3B‐II, a crucial step in autophagy initiation related to autophagosomes [51]. Treatment with the nanosystem efficiently altered this ratio, balancing it in favour of LC3B‐II (Figure 5B), which ratified autophagy induction by the nanosystem and might also have a key role in the mechanism of cell death. Moreover, the use of the autophagy inhibitor chloroquine (CQ), which blocks the fusion of autophagosomes with lysosomes at the final stages of autophagy [63], partially but significantly reversed the autophagy induced by MSN‐AP‐PdCur in TNBC cells (Figure 5C). The statistically significant decrease observed supports the involvement of autophagy in the cellular response to this nanosystem.

These findings demonstrate that MSN‐AP‐PdCur elicits a complex cellular response involving DNA damage, apoptosis and autophagy. The observed increase in phosphorylated H2AX suggests the induction of DNA double‐strand breaks, a form of genotoxic stress known to activate both apoptotic and autophagic pathways.

Consistent with this, the treatment with our nanosystem resulted in a significant rise in apoptotic cells, as determined by Annexin V staining. Importantly, this effect was partially reversed upon co‐treatment with the pan‐caspase inhibitor QVD, indicating that cell death is primarily caspase‐dependent. However, the incomplete rescue of cell viability implies the possible involvement of caspase‐independent pathways or partial caspase inhibition.

In parallel, the formation of autophagosomes observed in the flow cytometry assays was enhanced in response to the MSN‐AP‐PdCur, indicative of autophagy activation. Notably, co‐treatment with chloroquine—which blocks autophagosome—lysosome fusion—partially attenuated this response, although autophagic markers remained elevated relative to CQ untreated controls (Figure 5C). This suggests that autophagy is actively induced as a stress response to MSN‐AP‐PdCur. While initially cytoprotective, excessive or sustained autophagy could contribute to cell death, highlighting a potential interplay between autophagic and apoptotic mechanisms under stress conditions.

## 4 Conclusions

In this study, we demonstrated that the MSN‐AP‐PdCur nanomaterial designed successfully incorporated the curcumin–palladium complex, enabling a dose‐dependent delivery system for curcumin. This nanosystem showed good stability in a biological analogous medium, making it a suitable platform for drug encapsulation and controlled release. Moreover, the primary mechanism of action of the MSN‐AP‐PdCur nanosystem was the induction of autophagy, but it can also induce ROS production and the activation of apoptosis. These results show how the synthesised nanosystem activates different cell death pathways, which presents itself as a promising therapeutic tool in the treatment of TNBC.

However, the study is limited to in vitro findings. The absence of in vivo validation restricts the assessment of key parameters such as pharmacokinetics, biodistribution, systemic toxicity and overall antitumour efficacy in a physiologically relevant context. Therefore, future research should incorporate in vivo studies to more comprehensively evaluate the translational potential and clinical applicability of this nanosystem.

## Author Contributions

**Lorenzo Rivas‐García:** conceptualization (equal), formal analysis (equal), investigation (equal), visualization (equal), writing – original draft (equal). **José Manuel Méndez‐Arriaga:** formal analysis (equal), investigation (equal), visualization (equal), writing – original draft (equal). **Sandra Jiménez‐Falcao:** conceptualization (equal), investigation (equal). **Paula Sánchez‐Olivares:** investigation (equal), methodology (equal), visualization (equal). **Pablo Cristóbal‐Cueto:** investigation (equal), methodology (equal). **Alejandro Pinedo‐Serrano:** investigation (equal), methodology (equal). **Raquel López‐Rosa:** investigation (equal), methodology (equal). **Esther Sánchez‐López:** investigation (equal), methodology (equal). **Santiago Gómez‐Ruiz:** conceptualization (equal), funding acquisition (equal), methodology (equal), project administration (equal), resources (equal), supervision (equal), validation (equal), writing – review and editing (equal). **Eva M. Galán‐Moya:** conceptualization (equal), funding acquisition (equal), methodology (equal), project administration (equal), resources (equal), supervision (equal), validation (equal), writing – review and editing (equal).

## Conflicts of Interest

The authors declare no conflicts of interest.

## Data Availability

Under request.
